# Proton Bragg peak flash stereotactic radiosurgery for recurrent glioblastoma reirradiation^☆^

**DOI:** 10.1016/j.phro.2026.101044

**Published:** 2026-07-20

**Authors:** Yangguang Ma, Yuyu Wang, Tengda Zhang, Xingyi Zhao, Balaji Selvaraj, Chingyun Cheng, Michael Pennock, Jocelyn Jackson, Wei Guo, Xuanqin Mou, Benjamin Durkee, Jehee Isabelle Choi, Haibo Lin, Charles B. Simone, Minglei Kang, Hui Wu

**Affiliations:** a Radiotherapy Department, The First Affiliated Hospital of Zhengzhou University, Zhengzhou 450052, China; b Faculty of Medical Sciences, University of Groningen, University Medical Center Groningen, the Netherlands; c Department of Radiation Medicine, University of Wisconsin-Madison, Madison, WI 53792, USA; d New York proton center, New York, NY 10035, USA; e Department of Radiation Oncology, University of Pittsburgh Medical Center Hillman Cancer Center; University of Pittsburg Medical Center Beaver; Heritage Valley Health System, Pittsburgh, PA 15232, USA; f Radiation Oncology, the Affiliated Cancer, Hospital of Zhengzhou University & Henan Cancer Hospital, Zhengzhou 450003, China; g School of Information and Communications Engineering, Faculty of Electronic and Information Engineering, Xi'an Jiaotong University, Xi'an 710049, China

**Keywords:** reirradiation, recurrent glioblastoma, CyberKnife, Bragg peak FLASH, stereotactic radiosurgery

## Abstract

**Background and Purpose:**

Stereotactic Radiosurgery with robotic image-guided systems is promising for recurrent glioblastoma (rGBM), but long delivery times may increase morbidity and reduce accuracy. Proton single-energy Bragg-peak FLASH-RT (SEBP-FLASH) may address these limits by delivering each field in <1 s, with potential for normal-tissue sparing through the FLASH effect.

**Materials and Methods:**

The SEBP-FLASH technique was used to reoptimize treatment for rGBM patients treated with robotic image-guided systems. Patient-specific aperture devices were incorporated into the SEBP-FLASH technique for sharper penumbra. The gross tumor volume (GTV) coverage, conformity index (CI), dose gradient index (GI_50_ and GI_30_), and dose-volume histogram (DVHs), were evaluated. Additionally, ultra-high dose-rate ratios in critical organs-at-risk were assessed.

**Results:**

SEBP-FLASH successfully met all clinical objectives. Both SEBP-FLASH and robotic techniques showed comparable performance, with GI_50_ and GI_30_ values of 2.9 ± 0.5 and 6.2 ± 1.8 for SEBP-FLASH, versus 2.7 ± 0.2 and 5.5 ± 0.5 for robotic (*p*-values at 0.085 and 0.189). SEBP-FLASH demonstrated superior conformality, achieving a CI of 1.4 ± 0.2 compared to CK's 1.9 ± 0.3 (p-value 0.001). All other metrics were similar across both techniques. For the brain, the FLASH dose rate coverage (V_40 Gy/s_) exceeded 80% under a 5 Gy dose threshold and minimum monitor unit of 400.

**Conclusions:**

This study evaluated the dose-volume characteristics of SEBP-FLASH and robotic image-guided systems and found broadly comparable dose distributions, suggesting that SEBP-FLASH may be a feasible option for rGBM reirradiation. SEBP-FLASH also showed potential advantages in conformity, normal tissue sparing, and reduced on-table time.

## Highlights

• Bragg peak flash enables fractionated radiosurgery for recurrent glioblastoma.
• Compared to robotic surgery, the conformity index improved from 1.92 ± 0.30 to 1.43 ± 0.20.
• Treatment time was reduced from 1000 to 2 s for recurrent glioblastoma.
• Dose escalation in recurrent glioblastoma could be enabled by Bragg peak flash.

## Graphical abstract

**Figure:**
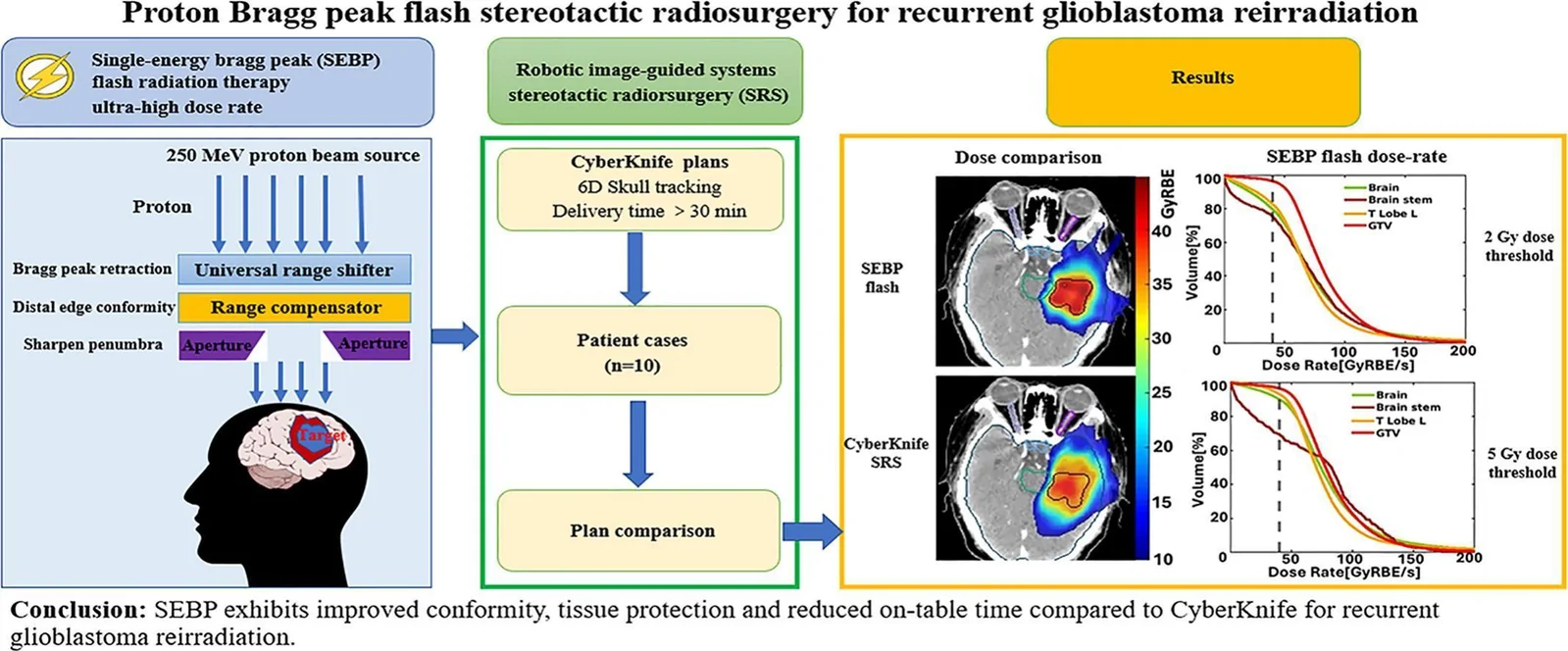

## 1 Introduction

Glioblastoma (GBM) is among the most aggressive brain cancers, with a median overall survival (OS) of 14 months and a 5-year survival rate of ∼7%. Approximately two-thirds of patients experience recurrence within two years of initial treatment [1], [2], [3]. Treatment options for recurrent GBM (rGBM) are limited. Without effective intervention, OS rarely exceeds 3–5 months. With active treatment, the median survival extends to approximately 7 months (ranging from 5 to 9.2 months), with 6-month progression-free survival (PFS) rates falling below 20% [4]. Preclinical and clinical evidence suggests the brain and spinal cord have considerable repair capacity, supporting reirradiation as a viable option for selected patients [5], [6], [7], [8], [9]. When conducting reirradiation, it is imperative to minimize the dose delivered to normal tissues surrounding the recurrent tumor. To fulfill this purpose, high-precision stereotactic techniques are an appealing option compared to conventional radiotherapy [10].

Salvage reirradiation is feasible for selected rGBM patients. Guidelines recommend reirradiation for younger patients with good performance status and a minimum interval of 6 months since initial radiotherapy [11], [12], [13], [14], [15], [16]. While salvage reirradiation is the primary treatment approach for rGBM, it can be associated with considerable side-effects and morbidity. Strictly limiting radiation doses to organs-of-interest (OOI) may compromise gross tumor volume (GTV) coverage. Balancing these considerations is challenging.

Stereotactic radiosurgery (SRS) has emerged as a prominent treatment modality in rGBM management and is increasingly accessible [17]. CyberKnife (CK), a robotic image-guided systems, can deliver highly conformal dose distribution through nonisometric noncoplanar beams of varying sizes, enabling steep dose gradients and effective OOI sparing [18]. Despite these advantages in dose distribution, its prolonged fraction delivery times (typical >30 min) pose significant challenges, as extended immobilization is physically and psychologically demanding, particularly for elderly or frail patients, and increases the risk of intrafraction motion, potentially compromising precision and treatment outcomes.

Proton therapy has shown promise for GBM, with some reports suggesting improved survival rates over conventional modalities [19]. In parallel, FLASH radiotherapy (FLASH-RT) has gained attention for its potential in reducing normal tissue side-effects while maintaining tumor control [20], [21], [22], particularly valuable in rGBM where tissue tolerance is already compromised. While early proton transmission beams studied achieved ultra-high dose rate (UHDR), they exposed distal OOIs to significant exit doses [23], [24]. To address this limitation and utilize the depth-dose characteristics of pencil beam scanning(PBS) protons, the Bragg peak FLASH (BP-FLASH) approach was proposed [25]. This technique aims to maximize the therapeutic gain by confining dose deposition to the tumor while minimizing exposure to surrounding tissues.

To date, no studies have explored BP-FLASH for rGBM treatment. We hypothesize this approach can address CK-based SRS (CK-SRS) limitations by offering rapid dose delivery with enhanced normal tissue sparing. Therefore, the aim of the study was to provide a comprehensive comparison of the dose-volume parameters between proton FLASH-RT and CK-SRS, alongside an analysis of UHDR characteristics within targets and OOIs to assess the feasibility and potential radiobiological advantages of FLASH-RT for rGBM.

## 2 Materials and methods

### 2.1 Patient selection and treatment plans

This IRB-approved retrospective study (AHEAD-AHNO2 2019199) included 10 rGBM patients with a solitary intracranial tumor, treated with fractionated CK-SRS between July 2022 and October 2023 (Table 1). Eligibility criteria required a minimum interval of 12 months from the initial radiotherapy course. GTV was delineated on planning CT fused with MRI, with a 2 mm margin added to define the planning target volume (PTV).

**Table 1:** The fractionation, target volume, diameter, and the time of tumor progression for all the ten rGBM patients treated using CK-SRS.

Patient No.	Fraction Dose (Gy)	Fractions	Target Volume (cm^3^)	Tumor Diameter (cm)	Time to Tumor Progression (month)
1	22	1	2.9	1.8	13
2	9	3	8.1	2.5	16
3	5	5	8.5	2.5	12
4	9	3	8.0	2.5	18
5	6	5	33.1	4.0	13
6	8	4	11.9	2.8	16
7	8	4	25.4	3.6	13
8	6	5	12.6	2.9	13
9	9	3	5.3	2.2	20
10	6	5	33.1	4.0	18

### 2.2 CyberKnife treatment planning

Clinical CK treatment plans were developed using Precision® treatment planning system (TPS, v1.1, Accuray, USA) and implemented on a CK-M6™ machine (C0533, Accuray, USA). The In-Cise™ MLC was used for plan optimization, together with 60–80 nodes, “Full Path” treatment anatomy and 6D Skull-tracking. Total monitor units (MUs) were not predefined, with an MU/node range set between 300 and 600, and beams delivering <10 MUs were excluded to reduce total MU. Four to eight dose-limiting shells were generated to improve conformity and dose falloff. Plan evaluation followed Radiation Therapy Oncology Group (RTOG)-0813, European Society for Radiotherapy and Oncology (ESTRO) guidance, and Timmerman dose guidance [26], [27], [28]. The GTV served as the target, and the conformity index (CI) and gradient index (GI) were evaluated following the RTOG definition [29]. GI_30_ and GI_50_ were computed and analyzed. Forward planning strategies aimed for minimum 95% PTV receiving prescribed dose and GI_50_ below 3. GI_50_ and GI_30_ defined as the ratios of the volumes receiving 50% and 30% of the prescribed dose to the prescription isodose volume.

### 2.3 Proton BP-FLASH treatment planning

Single-energy Bragg peak (SEBP) proton FLASH-RT utilizes a universal range shifter (URS) to retract the BP to the tumor target, with range compensators (RCs) further conforming the proton range to the target [30]. The distal fall-off (R_80_) of the 250 MeV BP is approximately 38 cm in water, with the range pullback calculated using a ray-tracing method (eq. 1). *WET(i)* represents the water-equivalent thickness at each designated location *i,* where the BP is intended to stop, calculated in the beam's eye view. The URS consists of six polymethyl methacrylate (PMMA) plastic plates of variable thickness, enabling range pullback from 34 to 0 cm with 1 cm increments. Mounted on the nozzle and rotating with the gantry, the URS and RC allow flexible air gap adjustments and multi-angle delivery. The URS and RC work together to align BPs with the target's distal edge (eq. 2).

(1) $$R\left(i\right)=R_{80}-\mathrm{WET}\left(i\right)$$

(2) $$\begin{array}{c} R\left(i\right)=D_{\mathrm{RC}}\left(i\right)+D_{\mathrm{URS}} \end{array}$$

Using a single fixed proton energy eliminates energy layer switching, enhancing dose rate per field and enabling achievement of the FLASH dose rate threshold (>40 Gy/s) [25]. Combining with multiple-field optimization (MFO), the approach enables optimization of beam number, gantry angles, PBS spot weights, and spot placements, achieving plan quality comparable to conventional intensity-modulated proton therapy (IMPT), as demonstrated in prior applications for lung [31], liver [32], head neck [33], prostate [34], breast [35], and spinal cord treatments [36].

**Fig. 1:**
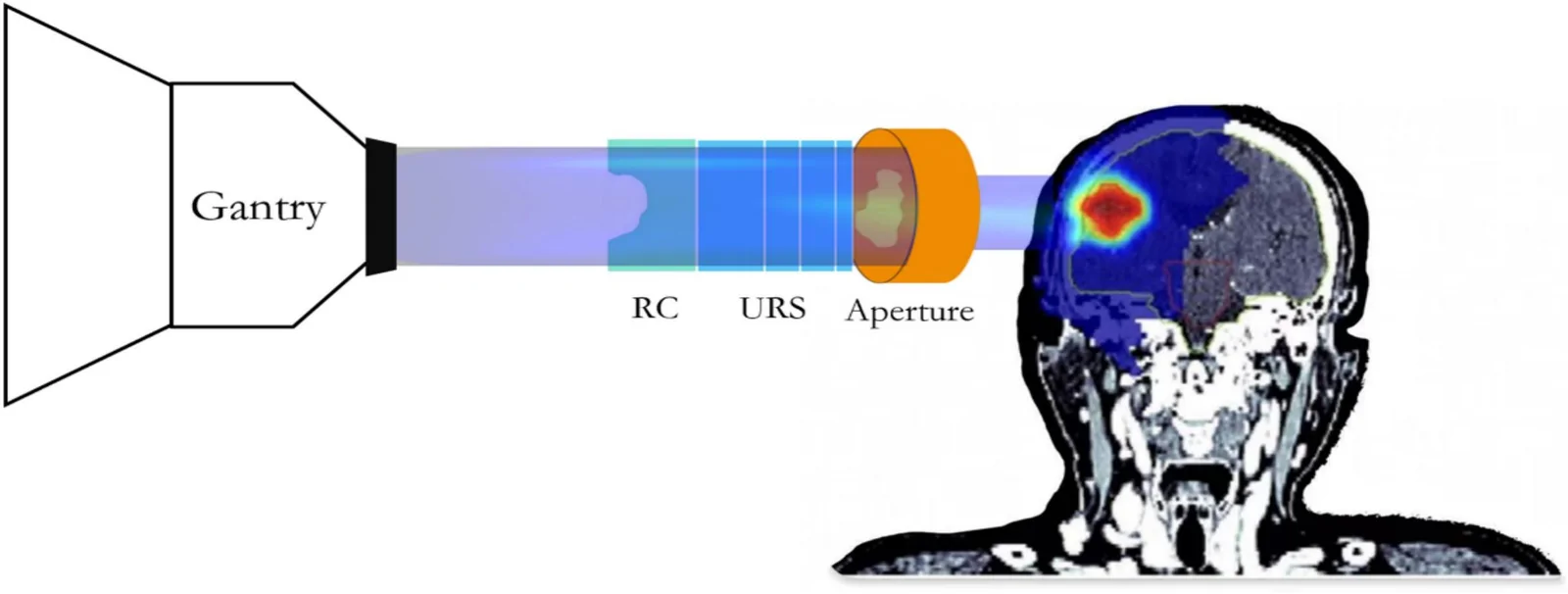
Depiction of the novel single-energy pristine Bragg peak (SEBP) FLASH technique for stereotactic radiosurgery (SRS) in recurrent glioblastoma (rGBM). The single-energy proton beam passes through the range compensator (RC) and universal range shifter (URS) to stop at the target's distal edge, while a beam-specific brass aperture sharpens the lateral dose falloff to minimize normal tissue exposure.

URS and RCs can increase the proton scanning spot size and result in greater lateral dose spread due to multiple Coulomb scattering [25]. Beyond a depth of 25 cm, the spot size increases to about twice its initial value [32]. This extra lateral scattering broadens the penumbra, risking unintended irradiation of adjacent normal tissues. To mitigate this effect, a patient-specific brass aperture was incorporated (Fig. 1). A previous study [37] demonstrated that such apertures can effectively limit lateral dose spread, protecting nearby OOIs such as the brainstem, spinal cord, and optic apparatus.

All FLASH-RT plans were optimized using an in-house–developed SEBP TPS based on the open-source toolkit matRad [38]. This system was commissioned using clinical beam parameters from the Varian ProBeam (Varian Medical Systems, USA) at the New York Proton Center. A fixed proton beam energy of 250 MeV was used, with one MU corresponding to approximately 5.29 × 10^6^ protons. Under FLASH mode, the minimum spot delivery time for a minimum MU was ∼2 ms [39]. Achieving FLASH dose rates required beam currents >100 nA at isocenter (compared to 2 nA conventional), which in turn imposed a minimum MU/spot requirement. To accommodate this limitation, spot map optimization strategy was applied to reduce low-MU spots while maintaining plan quality. All FLASH plans consisted of three or four beams with beam-specific apertures and RCs, with Minimum monitor unit (MMU) thresholds of 300 and 400 per spot, corresponding to isocenter beam currents of ∼127 and 169 nA, respectively.

Due to spot-by-spot delivery nature of PBS, FLASH-RT dose rate calculation requires voxel-level assessment rather than field-average approaches [39]. Among available methods, the PBS dose rate (PBSDR) [40] accounts for both spot delivery and scanning time, offering a conservative dose rate estimate in PBS-based FLASH treatments. This method was adopted to quantify FLASH coverage.

### 2.4 Plan quality and FLASH dose rate evaluation

Critical OOIs' dose metrics, along with the RTOG CI and GI for the GTV, were analyzed for both proton FLASH and CK plans. The Lyman-Kutcher-Burman (LKB) model was used to estimate normal tissue complication probability (NTCP) [41], [42], [43], [44]. The Lyman model [41] describes NTCP as a sigmoid function of dose, assuming a uniform dose distribution. To account for non-uniform irradiation, the effective volume method [42] converts heterogeneous doses into an equivalent uniform dose. When combined, this forms the LKB model [45]. NTCP parameters in our tool are based on QUANTEC [46]. A two-sided paired *t*-test assessed differences between plans (*p* < 0.05), with dose-volume parameters reported as mean ± standard deviation (SD) and median (1st–3rd quartile).

We used a conservative dose-rate estimation, evaluating dose-rates on a field-by-field basis [47]. For FLASH plans, voxel-based UHDR analyses were performed, calculating the normal brain volume receiving ≥40 Gy/s (V_40 Gy/s_). Here, dose-rate analysis was limited to normal brain tissue because it is traversed by all treatment beams, encompasses a much larger volume than other critical structures, and represents a primary consideration in treatment plan optimization. Since the FLASH sparing effect depend on both high dose-rate and a dose threshold—although the exact value remains under investigation—V_40 Gy/s_ was evaluated using thresholds of 2 and 5 Gy, with higher thresholds yielding more conservative FLASH ratio estimate [47]. Because most *in vivo* FLASH biological experiments employ single-beam configuration [36], [48], V_40 Gy/s_ was calculated per field, defined as the fraction of voxels within a ROI receiving a dose above the given threshold d*(2 or 5 Gy) and a dose-rate exceeding 40 Gy/s, relative to all voxels receiving a dose above d* from a single beam, as expressed in eq. (3).

(3) $$V_{40\;\mathrm{Gy}/s}=\frac{\sum_{i=1}^{N}\sum_{j=1}^{M}I_{\mathrm{ij}}^{40}}{\sum_{i=1}^{N}\sum_{j=1}^{M}I_{\mathrm{ij}}}$$

where:

$$I_{\mathrm{ij}}^{40}=\left\{\begin{array}{c} 1,\text{if}\;D_{\mathrm{ij}}>d^{*}\;\text{and}\;{\dot{D}}_{\mathrm{ij}}>40\;\mathrm{Gy}/s\\ 0,\text{otherwise}\; \end{array}\right)$$

$$I_{\mathrm{ij}}=\left\{\begin{array}{c} 1,\;\text{if}\;D_{\mathrm{ij}}>d^{*}\\ 0,\;\text{otherwise}\; \end{array}\right)$$

Definitions $I_{\mathrm{ij}}^{40}=$ indicator function for voxels meeting both the dose threshold d^⁎^ and dose-rate threshold of 40 Gy/s, I_ij_ = indicator function for voxels meeting only the dose threshold d^⁎^, i = field index (1 ≤ i ≤ N); j = voxel index (1 ≤ j ≤ M), N = number of fields, M = number of voxels within the ROI, d^⁎^ = dose threshold (2 or 5 Gy in this study), D_ij_ = dose to the j-th voxel from the i-th field, ${\dot{D}}_{\mathrm{ij}}$= dose-rate of the j-th voxel from the i-th field.

## 3 Results

**Fig. 2:**
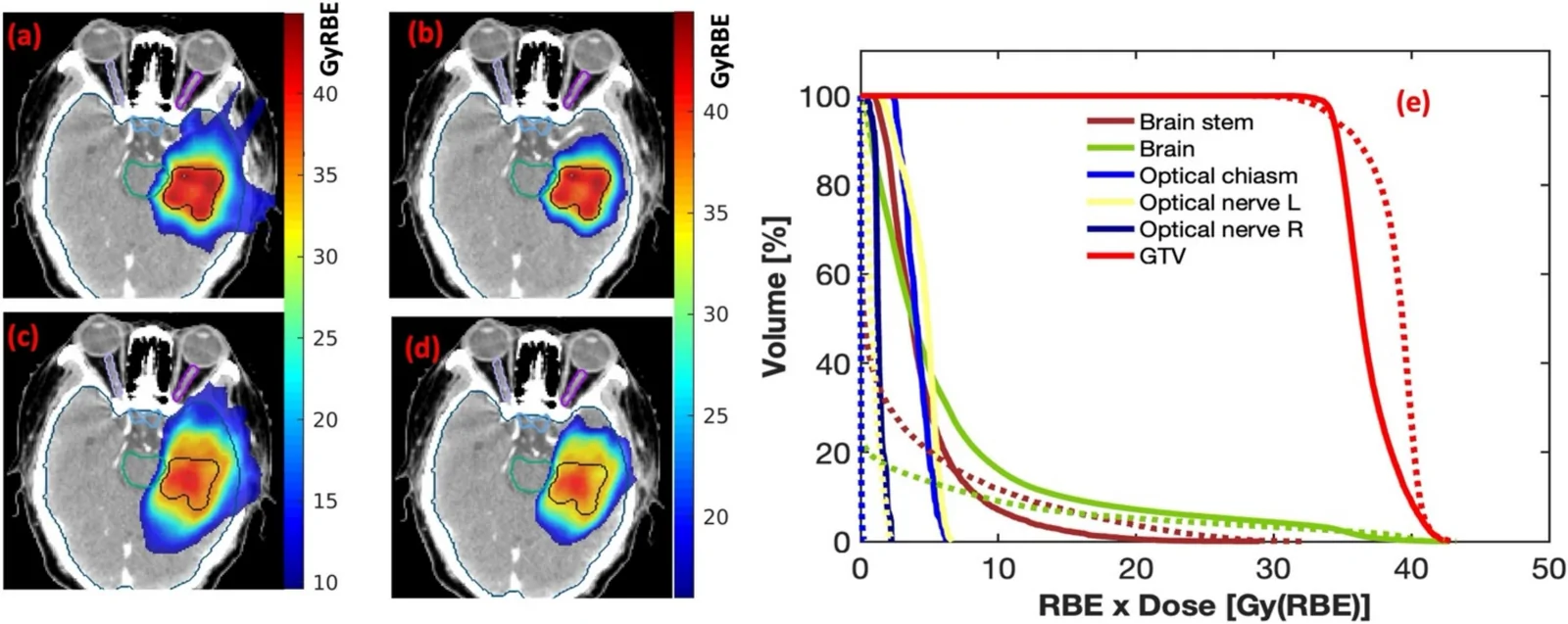
Comparison of dose-volume parameters between SEBP FLASH and CyberKnife (CK) stereotactic radiosurgery (SRS) for a representative patient:(a-b) SEBP FLASH SRS dose distribution with 30% and 50% isodose lines, (c-d) CK-SRS dose distribution with 30% and 50% isodose lines; (e) Dose-Volume Histogram (DVH) comparison between CK (solid) and SEBP FLASH (dotted) plans.

SEBP FLASH achieved superior coverage and isodose conformity compared to CK, with smaller 30% and 50% isodose volumes (Fig. 2). SEBP FLASH and CK plans showed comparable brainstem, optic nerves, GI_50_, and GI_30_ metrics, with no statistically significant difference(*p* > 0.05, Table2). Specifically, SEBP outperformed CK in optic chiasm maximum dose (17.1 ± 40.7% vs. 25.2 ± 37.0%, *p* < 0.01) and CI (1.4 ± 0.2 vs. 1.9 ± 0.3, p < 0.01), though GTV D_max_ was higher (146.6 ± 13.2% vs. 131.1 ± 12.8%, p < 0.01). NTCP values were comparable across all evaluated OOIs. Therefore, disregarding the potential FLASH biological sparing effects, the dose-volume parameters of the two techniques were comparable (Table 2).

**Table 2:** Comparison of dose-volume parameters between CK and SEBP FLASH plans.

Comparison parameters	SEBP FLASH plan		CK plan		p-value
	Mean ± SD	Median (Q1-Q3)	Mean ± SD	Median (Q1-Q3)	
Brain stem D_max_*	32.4 ± 52.4%	13.1% (1.0–16.1)	32.1 ± 37.4%	14.6% (6.1–57.0)	0.96
Chiasm D_max_	17.1 ± 40.7%	6.2% (0–12.7)	25.2 ± 37.0%	4.9% (0.4–18.0)	<0.01
Optic nerves D_max_	7.0 ± 19.0%	0.5% (0.0–8.3)	10.2 ± 7.2%	8.4% (0.1–14.7)	0.59
GTV D_max_	146.6 ± 13.2%	138.0% (118.8–143.7)	131.1 ± 12.8%	145.7% (138.9–152.0)	<0.01
GI_50_	2.9 ± 0.5	2.9 (2.8–3.2)	2.7 ± 0.2	2.6 (2.7–2.7)	0.09
GI_30_	6.2 ± 1.8	6.2 (5.5–6.7)	5.5 ± 0.5	5.2 (4.6–5.6)	0.19
CI	1.4 ± 0.2	1.8 (1.3–2.0)	1.9 ± 0.3	1.6 (1.4–1.7)	<0.01
Brain stem NTCP	0.0 ± 0.0	0	0.0 ± 0.0	0	0.29
Brain NTCP	0.0 ± 0.0	0.1 (0.1–0.1)	0.0 ± 0.0	0.2 (0.0–0.3)	0.37
Chiasm NTCP	0.1 ± 0.2	0	0.1 ± 0.2	0	0.34
Optic nerves NTCP	0.0 ± 0.0	0	0.0 ± 0.0	0	0.35

* All dose values (D_max_) are expressed as percentages of the prescription dose. SD, Q1, and Q3 represent standard deviation, first quartile, and third quartile, respectively. CI (conformity index), GI (gradient index) and NTCP (normal tissue complication probability) are unitless parameters.

**Fig. 3:**
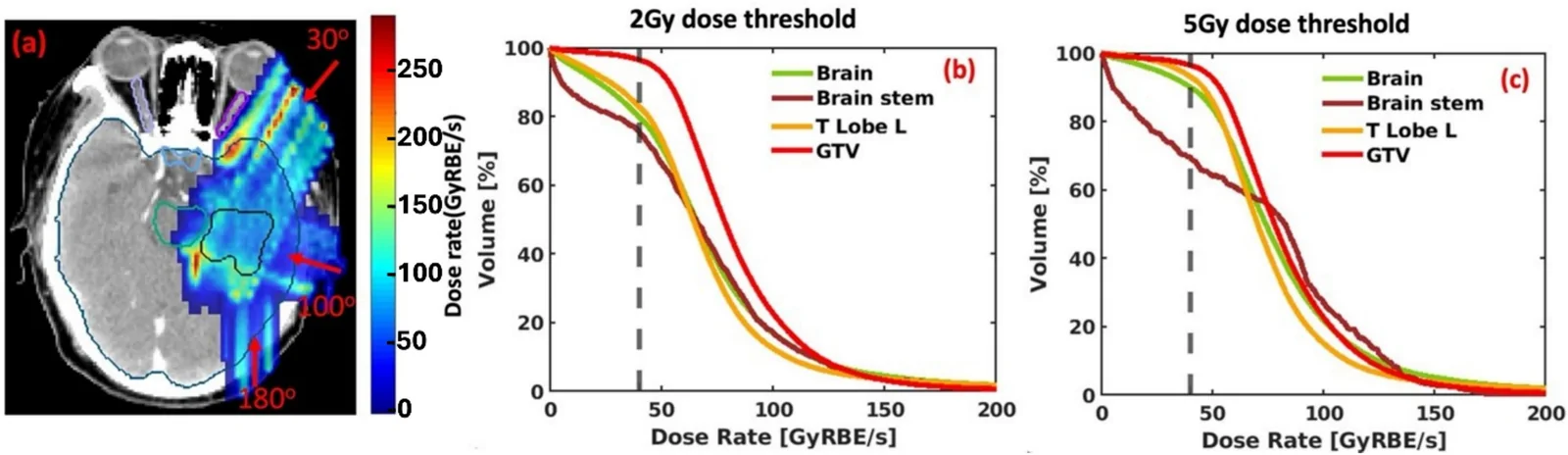
Dose-rate distribution for the same representative patient. (a) 2D dose-rate maps for beams at gantry angles 30°, 100°, and 180°. (b–c) Dose-rate volume histograms (DRVHs) for OOIs and target at dose thresholds of 2 and 5 Gy. Vertical dashed lines indicate the 40 Gy/s threshold.

The dose-rate distribution of SEBP FLASH was spatially heterogeneous, with high-dose-rate strips corresponding to individual proton spot, as illustrated in a representative case (Fig. 3a). Increasing the dose threshold from 2 to 5 Gy increased the FLASH ratio for the brain and left temporal lobe (Figs. 3(b and c)). Under both thresholds, SEBP FLASH achieved substantial FLASH dose-rate coverage of the brain and GTV across MMU settings of 300 and 400 (Table 3).

**Table 3:** FLASH dose rate coverage (V_40 Gy/s_) of brain and GTV under different MMU guidance and dose thresholds (2 Gy and 5 Gy).

MMU*	Organ	Dth** = 2 Gy		Dth = 5 Gy	
		Mean ± SD	Median (Q1-Q3)	Mean ± SD	Median (Q1-Q3)
300	Brain	55.3% ± 13.9%	53.6% (47.4–66.3)	81.0% ± 14.7%	81.4% (77.2–91.4)
	GTV	92.5% ± 9.8%	96.1% (91.2–99.6)	92.5% ± 9.8%	96.1% (91.2–99.7)
400	Brain	60.5% ± 14.8%	60.4% (47.9–70.8)	85.8% ± 13.8%	88.7% (80.4–96.4)
	GTV	95.8% ± 6.8%	99.0% (95.2–100)	95.8% ± 6.8%	99.0% (95.2–100)

* MMU = minimum monitor unit; ** Dth = dose threshold; SD, Q1, and Q3 represent Standard Deviation, First Quartile, and Third Quartile, respectively.

Increasing MMU from 300 to 400 improved FLASH dose-rate coverage, similarly, increasing the dose threshold from 2 to 5 Gy also increased V_40 Gy/s_ (Table 3). Each FLASH field required approximately 0.47 ± 0.15 s at MMU 400 and 0.62 ± 0.23 s at MMU 300, corresponding to total delivery time of 1.41 and 1.86 s per three-field plan, respectively. In comparison, the mean CyberKnife delivery time was 1020.42 ± 584.30 s.

## 4 Discussion

SEBP FLASH is an emerging radiotherapy technique that delivers highly conformal dose distributions using range shifters and compensators to position the BP at the distal target edge. This study assessed the dose-volume parameters performance of SEBP FLASH as a potential alternative to CK-based SRS for the treatment of rGBM in a hypofractionation regimen. SEBP achieved comparable dose-volume parameters performance to CK with adequate target coverage, while demonstrating a reduced low-dose bath in surrounding brain tissue owing to the characteristic physical properties of protons. However, SEBP showed slightly inferior dose uniformity, likely attributable to the limited degrees of freedom inherent to single-energy beam delivery. Overall, these findings suggested that SEBP FLASH represented a technically feasible approach that offers dosimetric advantages for this challenging clinical setting.

CI was a critical differentiator, as precise conformity is essential in brain SRS, particularly in the setting of reirradiation, where normal brain tissue had already received prior radiotherapy dose [49]. In this study, SEBP achieved significantly improved CI over CK (1.4 vs.1.9, *p* < 0.01), suggesting superior normal tissue sparing, which was particularly important in high-dose single-fraction treatments. In contrast, GI_50_ and GI_30_ showed no significant differences between the two techniques, indicating that patient-specific brass apertures in SEBP effectively mitigated lateral dose spread. These findings indicated that SEBP FLASH achieved comparable dose distribution to CK with improved conformity and reduced normal tissue exposure. Combined with significant short treatment time, SEBP provided meaningful clinical advantages for rGBM reirradiation, even without considering the potential FLASH sparing effect. Patient-specific brass apertures in SEBP FLASH delivery may introduce geometric uncertainties, particularly in multi-fraction settings. Since the apertures were generated by expanding the field spot map contour, similar to conventional photon collimators, position verification, image guidance, and adaptive planning were needed for safe and accurate delivery.

Beyond favorable dose-volume parameter characteristics, SEBP could deliver UHDR potentially triggering the FLASH sparing effect. FLASH coverage was evaluated using the PBSDR method, which unlike dose-averaged dose-rate (DADR) [39], PBSDR accounts for both spot delivery and scanning dynamics, providing a more temporally accurate and conservative voxel level dose-rate estimate.

In this study, only normal brain tissue was included in the dose-rate analysis because it represents a much larger volume than other critical structures (e.g., brainstem optic nerves) and received substantial dose from each FLASH field. In contrast, small OOIs can often be spared through beam arrangement. If the target was sufficiently separated from the brainstem, brainstem dose may be fully avoided. Since the FLASH effect was dose-dependent [50], evaluating dose-rate in minimally irradiated structures was not clinically meaningful. Therefore, normal brain tissue provided a more relevant metric for assessing the effect of machine parameters on potential FLASH benefit. Furthermore, as all SEBP FLASH plans met clinical dose guidance (Table 2), often outperforming CK plans, the inability to achieve FLASH dose rates in structures such as the brainstem is not a clinical concern, given that doses remain within safe limits. These findings are consistent with previous reports comparing SE-BP FLASH and IMPT, which similarly demonstrated faster delivery with comparable dose distributions and OOI sparing, even without accounting for the FLASH effect [25], [51],collectively underscoring the substantial clinical potential of SE-BP FLASH.

As shown in Table 3, both higher MMU and an elevated dose threshold (2 to 5 Gy) increased V_40 Gy/s_. Higher MMU increased per-spot dose-rate [39] and reduced low-weight spots, but at the cost of reduced modulation flexibility, which may partly explain the higher D_max_ and lower dose homogeneity in SEBP plans. In contrast, a higher dose threshold increased V_40 Gy/s_ by restricting analysis to inherently higher-dose-rate voxels, without changing beam delivery. Given the relatively small sample size, both mean ± SD and median (Q1–Q3) were reported in Table 2 and Table 3: the former reflects average magnitude and variability, while the latter is less sensitive to potential outliers.

No consensus currently exists on the dose-rate threshold for the FLASH effect, which may vary by tissue, and V_40 Gy/s_ therefore had inherent limitations. Current evidence suggested a dose threshold of approximately 4–10 Gy for FLASH activation [52], [53], [54], [55]. In this study, 2 and 5 Gy were selected to represent low- and intermediate-dose regions. Applied at the individual-field level, these thresholds provided a conservative framework to assess whether SEBP can maintain meaningful FLASH coverage under the prescription and fractionation conditions used here, even if a higher minimum dose threshold is ultimately required.

Importantly, V_40 Gy/s_ coverage varied across structures, with the brain showing the most consistent and extensive UHDR coverage, exceeding 80% under high MMU and dose-threshold conditions. This was particularly significant given that normal brain tissue in rGBM reirradiation was often near tolerance, limiting safe dose escalation and potentially compromising local control [49], [56]. High FLASH coverage raised the possibility of exploiting the FLASH sparing effect to overcome this constraint. Preclinical evidence in mouse brain models suggested reduced side-effects with FLASH: whole-brain irradiation preserved cognitive function at FLASH dose rates, and glioblastoma models showed less normal brain side-effects than conventional proton therapy [57]. Recent studies described this sparing effect using the dose-modifying factor (DMF), which for the central nervous system was ∼1.4 [22], [58], [59], [60]. This suggested SEBP could enable tumor dose escalation while maintaining equivalent normal tissue side-effects compared to CK-SRS. This could improve local control in reirradiation, supported by a large multicenter study linking higher reirradiation doses to improved survival in rGBM [61].

It should be noted that the target volumes evaluated in this study were relatively small, consistent with the typical clinical use of CK reirradiation [56], but may not be representative of the full spectrum of patients with recurrent GBM. The feasibility and potential clinical benefit of the SEBP FLASH approach for larger target volumes remain to be established. In particular, treatment of larger volumes may introduce greater technical challenges in achieving UHDR while maintaining acceptable dose conformality and normal tissue sparing.

In summary, our analysis suggested that SEBP can achieve high quality distributions while delivering UHDR to surrounding normal tissues under clinically feasible conditions, especially with appropriate MMU thresholds. The combination of improved conformity, shorter treatment time, and substantial FLASH dose-rate coverage indicated that SEBP may be a promising approach for rGBM reirradiation, where normal tissue tolerance was limited. Using a FLASH-capable, single-energy proton beam, this study systematically compared the dose-volume parameters and evaluated the dose-rate characteristics of SEBP and found that both could be favorably optimized. These results provide useful groundwork for future clinical translation; importantly, further preclinical validation and prospective clinical studies were essential to clarify the therapeutic potential of FLASH-RT in glioblastoma.

## Declaration of competing interest

The authors declare that they have no known competing financial interests or personal relationships that could have appeared to influence the work reported in this paper.
